# *N*,*N*′-Bis[3,5-bis­(2,6-diisopropyl­phen­yl)phen­yl]butane-2,3-diimine

**DOI:** 10.1107/S1600536811031254

**Published:** 2011-08-11

**Authors:** Tracy L. Lohr, Warren E. Piers, Masood Parvez

**Affiliations:** a Department of Chemistry, The University of Calgary, 2500 University Drive NW, Calgary, Alberta, Canada T2N 1N4

## Abstract

The title mol­ecule, C_64_H_80_N_2_, lies on an inversion center wherein the central butane­diimine fragment [N=C(Me)—C(Me)=N] is essentially planar [maximum deviation = 0.002 (2) Å] and its mean plane forms a dihedral of 70.88 (10)° with the attached benzene ring. In the symmetry-unique part of the mol­ecule, the dihedral angles between the benzene ring bonded to the N atom and the other two benzene rings are 89.61 (6) and 82.77 (6)°.

## Related literature

For background to water splitting, see: Yang & Hall (2010 ▶); Kee *et al.* (2011 ▶); Blakemore *et al.* (2010 ▶). For related structures, see: Ionkin & Marshall (2004 ▶); Zou *et al.* (2008 ▶); Lohr *et al.* (2011 ▶).
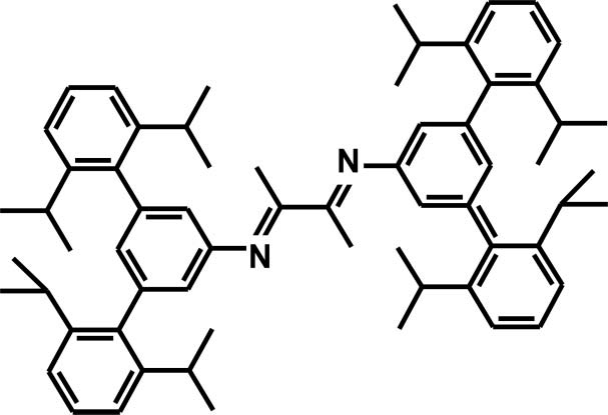


## Experimental

### 

#### Crystal data

C_64_H_80_N_2_
*M* *_r_* = 877.30
Triclinic, 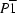

*a* = 8.512 (3) Å
*b* = 11.513 (3) Å
*c* = 16.501 (6) Å
α = 101.456 (18)°
β = 97.471 (13)°
γ = 99.505 (17)°
*V* = 1540.8 (9) Å^3^
*Z* = 1
Mo *K*α radiation
μ = 0.05 mm^−1^
*T* = 173 K
0.16 × 0.14 × 0.06 mm

#### Data collection

Nonius KappaCCD diffractometer with Bruker APEXII CCD detector
Absorption correction: multi-scan (*SORTAV*; Blessing, 1997 ▶) *T*_min_ = 0.992, *T*_max_ = 0.997
10648 measured reflections
5610 independent reflections
4274 reflections with *I* > 2σ(*I*)
*R*_int_ = 0.028

#### Refinement

*R*[*F* ^2^ > 2σ(*F* ^2^)] = 0.069
*wR*(*F* ^2^) = 0.197
*S* = 1.06
5610 reflections
307 parameters
H-atom parameters constrained
Δρ_max_ = 0.35 e Å^−3^
Δρ_min_ = −0.26 e Å^−3^

### 

Data collection: *COLLECT* (Hooft, 1998 ▶); cell refinement: *DENZO* (Otwinowski & Minor, 1997 ▶); data reduction: *SCALEPACK* (Otwinowski & Minor, 1997 ▶); program(s) used to solve structure: *SHELXS97* (Sheldrick, 2008 ▶); program(s) used to refine structure: *SHELXL97* (Sheldrick, 2008 ▶); molecular graphics: *ORTEP-3 for Windows* (Farrugia, 1997 ▶); software used to prepare material for publication: *SHELXL97*.

## Supplementary Material

Crystal structure: contains datablock(s) global, I. DOI: 10.1107/S1600536811031254/lh5297sup1.cif

Structure factors: contains datablock(s) I. DOI: 10.1107/S1600536811031254/lh5297Isup2.hkl

Supplementary material file. DOI: 10.1107/S1600536811031254/lh5297Isup3.cml

Additional supplementary materials:  crystallographic information; 3D view; checkCIF report

## Figures and Tables

**Fig. 1.**
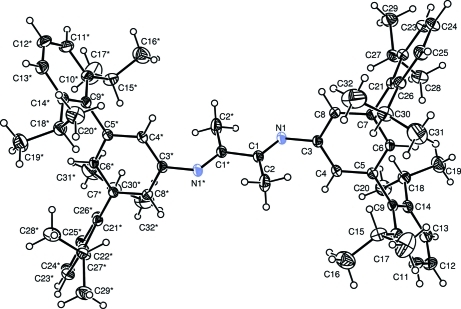
The title molecule with displacement ellipsoids plotted at 30% probability level (Farrugia, 1997). Primed atoms are related by the symmetry code (-x, -y+1, -z).

## supplementary crystallographic information

### Comment

To meet the ever-growing demand for green and carbon neutral energy, water splitting for the generation of hydrogen fuel represents an appealing strategy. To do their part, chemists have been looking at the steps towards organometallic mono-nuclear water splitting. (Yang & Hall, 2010; Kee *et al.*, 2011; Blakemore *et al.*, 2010). Our group is currently exploring the usage of platinum to mediate this reaction in an effort to understand the fundamental steps of O—H and O—O bond activation. This research is directed at synthesizing and studying plausible intermediates (Pt—OH and Pt—H species) in order to determine what role they play in the water activation process. The title compound was synthesized as a ligand to stabilize and isolate these highly reactive monomeric species for further mechanistic study.

In the title compound (Fig. 1), the central butanediimine fragment (N═ C(Me)–C(Me)═N) is essentially planar (maximum deviation of C1 being 0.002 (2) Å). The benzene ring (C3–C8) lies at 70.88 (10)° with respect to the mean-plane of the butanediimine fragment. The dihedral angles between the benzene ring bonded to N1 and benzene rings C9–C14 and C21–C26 are 89.61 (6) and 82.77 (6)°, respectively. The molecular dimesions in the title compound agree very well with the corresponding molecular dimensions reported in a few closely related compounds (Ionkin & Marshall, 2004; Zou *et al.*, 2008; Lohr *et al.*, 2011).

### Experimental

Synthesis of (ArN=C(Me)—C(Me)=NAr) (Ar = 3,5-bis(2,6-diisopropylphenyl)benzene): In air, 3,5-bis(2,6-diisopropylphenyl)aniline (0.190 g, 0.471 mmol) and 2,3-butadione (0.021 ml, 0.236 mmol) were dissolved in MeOH (35 ml). To this yellow solution were added 3 drops of formic acid and the mixture was stirred at room temperature and a bright yellow precipitate formed overnight. The mixture was stirred for 14 h, cooled, filtered, washed with cold MeOH (3 *x* 5 ml), and dried over an aspirator for 3 h. The title compound was isolated as a light yellow solid (0.183 g, 45%). X-ray quality crystals were obtained through slow cooling to 243 K in concentrated ethyl acetate.

### Refinement

Though the H-atoms were visible in the difference electron density maps they were included at geometrically idealized positions with C—H = 0.95, 0.98 and 1.00 Å for aryl, methine and methyl type H-atoms, respectively. The H-atoms were assigned *U*_iso_ = 1.2 times *U*_eq_(C).

### Crystal data

**Table:**

C_64_H_80_N_2_	*Z* = 1
*M**_r_* = 877.30	*F*(000) = 478
Triclinic, *P*1	*D*_x_ = 0.945 Mg m^−^^3^
Hall symbol: -P 1	Mo *K*α radiation, λ = 0.71073 Å
*a* = 8.512 (3) Å	Cell parameters from 6423 reflections
*b* = 11.513 (3) Å	θ = 1.0–25.4°
*c* = 16.501 (6) Å	µ = 0.05 mm^−^^1^
α = 101.456 (18)°	*T* = 173 K
β = 97.471 (13)°	Prism, pale yellow
γ = 99.505 (17)°	0.16 × 0.14 × 0.06 mm
*V* = 1540.8 (9) Å^3^	

### Data collection

**Table:**

Nonius KappaCCD diffractometer with Bruker APEXII CCD detector	5610 independent reflections
Radiation source: fine-focus sealed tube	4274 reflections with *I* > 2σ(*I*)
graphite	*R*_int_ = 0.028
ω and φ scans	θ_max_ = 25.4°, θ_min_ = 1.8°
Absorption correction: multi-scan (*SORTAV*; Blessing, 1997)	*h* = −10→10
*T*_min_ = 0.992, *T*_max_ = 0.997	*k* = −12→13
10648 measured reflections	*l* = −19→19

### Refinement

**Table:**

Refinement on *F*^2^	Primary atom site location: structure-invariant direct methods
Least-squares matrix: full	Secondary atom site location: difference Fourier map
*R*[*F*^2^ > 2σ(*F*^2^)] = 0.069	Hydrogen site location: inferred from neighbouring sites
*wR*(*F*^2^) = 0.197	H-atom parameters constrained
*S* = 1.06	*w* = 1/[σ^2^(*F*_o_^2^) + (0.0937*P*)^2^ + 0.8313*P*] where *P* = (*F*_o_^2^ + 2*F*_c_^2^)/3
5610 reflections	(Δ/σ)_max_ = 0.003
307 parameters	Δρ_max_ = 0.35 e Å^−^^3^
0 restraints	Δρ_min_ = −0.26 e Å^−^^3^

### Special details

**Table:**

Experimental. ^1^H NMR (400 MHz, CDCl_3_): δ = 1.08 (d, 24H, CH(C*H*_3_)_2_), 1.15 (d, 24H, CH(C*H*_3_)_2_), 2.23 (s, 6H, N═C–C*H*_3_), 2.84 (m, 8H, C*H*(CH_3_)_2_), 6.62 (d, 4H, Ar–H), 6.79 (t, 2H, Ar–H), 7.21 (d, 8H, Ar-H), 7.34 (t, 4H, Ar–H). ^13^C NMR (100 MHz, CDCl_3_): δ = 15.64 (N═C–*C*H_3_), 24.31 (CH(*C*H_3_)_2_), 24.44 (CH(*C*H_3_)_2_), 30.65 (*C*H(CH_3_)_2_), 118.09 (Ar–CH), 122.71 (Ar–CH), 126.91 (Ar–CH), 128.09 (Ar–CH), 139.08 (Ar–C), 141.66 (Ar–C), 146.84 (Ar–C), 150.89 (Ar–C), 169.06 (N═*C*–CH_3_).
Geometry. All e.s.d.'s (except the e.s.d. in the dihedral angle between two l.s. planes) are estimated using the full covariance matrix. The cell e.s.d.'s are taken into account individually in the estimation of e.s.d.'s in distances, angles and torsion angles; correlations between e.s.d.'s in cell parameters are only used when they are defined by crystal symmetry. An approximate (isotropic) treatment of cell e.s.d.'s is used for estimating e.s.d.'s involving l.s. planes.
Refinement. Refinement of *F*^2^ against ALL reflections. The weighted *R*-factor *wR* and goodness of fit *S* are based on *F*^2^, conventional *R*-factors *R* are based on *F*, with *F* set to zero for negative *F*^2^. The threshold expression of *F*^2^ > σ(*F*^2^) is used only for calculating *R*-factors(gt) *etc*. and is not relevant to the choice of reflections for refinement. *R*-factors based on *F*^2^ are statistically about twice as large as those based on *F*, and *R*- factors based on ALL data will be even larger.

### Fractional atomic coordinates and isotropic or equivalent isotropic displacement parameters (Å^2^)

**Table:**

	*x*	*y*	*z*	*U*_iso_*/*U*_eq_	
N1	0.0422 (2)	0.46436 (15)	0.09717 (11)	0.0335 (4)	
C1	0.0718 (2)	0.49926 (18)	0.03123 (12)	0.0303 (5)	
C2	0.2367 (3)	0.5377 (3)	0.01107 (16)	0.0541 (7)	
H2A	0.2353	0.5091	−0.0491	0.065*	
H2B	0.3152	0.5032	0.0429	0.065*	
H2C	0.2674	0.6261	0.0262	0.065*	
C3	0.1702 (2)	0.46292 (18)	0.16106 (12)	0.0295 (4)	
C4	0.2616 (2)	0.56921 (18)	0.21263 (13)	0.0307 (5)	
H4	0.2407	0.6449	0.2042	0.037*	
C5	0.3836 (2)	0.56590 (17)	0.27654 (12)	0.0286 (4)	
C6	0.4108 (2)	0.45436 (17)	0.28880 (12)	0.0278 (4)	
H6	0.4941	0.4515	0.3323	0.033*	
C7	0.3184 (2)	0.34646 (17)	0.23857 (12)	0.0272 (4)	
C8	0.1969 (2)	0.35137 (18)	0.17475 (12)	0.0291 (4)	
H8	0.1322	0.2787	0.1405	0.035*	
C9	0.4870 (2)	0.68017 (17)	0.33082 (13)	0.0317 (5)	
C10	0.6300 (3)	0.73029 (18)	0.30592 (14)	0.0374 (5)	
C11	0.7271 (3)	0.8346 (2)	0.35794 (17)	0.0466 (6)	
H11	0.8251	0.8689	0.3425	0.056*	
C12	0.6828 (3)	0.8886 (2)	0.43142 (17)	0.0519 (7)	
H12	0.7500	0.9602	0.4658	0.062*	
C13	0.5419 (3)	0.8399 (2)	0.45557 (15)	0.0468 (6)	
H13	0.5125	0.8787	0.5062	0.056*	
C14	0.4423 (3)	0.73418 (19)	0.40628 (13)	0.0370 (5)	
C15	0.6785 (3)	0.6753 (2)	0.22388 (16)	0.0466 (6)	
H15	0.6063	0.5943	0.2010	0.056*	
C16	0.6502 (5)	0.7543 (3)	0.1599 (2)	0.0793 (10)	
H16A	0.6741	0.7153	0.1058	0.095*	
H16B	0.7214	0.8339	0.1802	0.095*	
H16C	0.5373	0.7639	0.1532	0.095*	
C17	0.8506 (4)	0.6567 (4)	0.2347 (3)	0.0946 (12)	
H17A	0.8753	0.6198	0.1802	0.114*	
H17B	0.8642	0.6033	0.2734	0.114*	
H17C	0.9240	0.7348	0.2574	0.114*	
C18	0.2893 (3)	0.6814 (2)	0.43511 (14)	0.0420 (5)	
H18	0.2390	0.6038	0.3939	0.050*	
C19	0.3249 (4)	0.6521 (3)	0.52078 (18)	0.0652 (8)	
H19A	0.2246	0.6125	0.5352	0.078*	
H19B	0.3705	0.7269	0.5631	0.078*	
H19C	0.4025	0.5979	0.5193	0.078*	
C20	0.1674 (4)	0.7648 (3)	0.4347 (2)	0.0641 (8)	
H20A	0.0657	0.7246	0.4474	0.077*	
H20B	0.1480	0.7835	0.3793	0.077*	
H20C	0.2103	0.8398	0.4773	0.077*	
C21	0.3505 (2)	0.22790 (17)	0.25381 (13)	0.0299 (5)	
C22	0.2565 (3)	0.16664 (18)	0.30220 (14)	0.0350 (5)	
C23	0.2938 (3)	0.0592 (2)	0.31816 (15)	0.0437 (6)	
H23	0.2319	0.0170	0.3511	0.052*	
C24	0.4189 (3)	0.0126 (2)	0.28706 (16)	0.0487 (6)	
H24	0.4431	−0.0604	0.2991	0.058*	
C25	0.5085 (3)	0.0721 (2)	0.23850 (16)	0.0458 (6)	
H25	0.5936	0.0389	0.2168	0.055*	
C26	0.4766 (3)	0.18020 (18)	0.22058 (14)	0.0361 (5)	
C27	0.1190 (3)	0.2182 (2)	0.33720 (16)	0.0427 (6)	
H27	0.0728	0.2616	0.2958	0.051*	
C28	0.1793 (3)	0.3116 (2)	0.42019 (17)	0.0512 (6)	
H28A	0.0904	0.3498	0.4371	0.061*	
H28B	0.2673	0.3734	0.4130	0.061*	
H28C	0.2189	0.2714	0.4636	0.061*	
C29	−0.0188 (3)	0.1216 (3)	0.3474 (2)	0.0591 (7)	
H29A	−0.1114	0.1585	0.3595	0.071*	
H29B	0.0173	0.0855	0.3938	0.071*	
H29C	−0.0507	0.0587	0.2954	0.071*	
C30	0.5731 (3)	0.2413 (2)	0.16369 (16)	0.0451 (6)	
H30	0.5383	0.3200	0.1632	0.054*	
C31	0.7519 (4)	0.2698 (4)	0.1956 (3)	0.1020 (15)	
H31A	0.8079	0.3124	0.1585	0.122*	
H31B	0.7908	0.1945	0.1968	0.122*	
H31C	0.7738	0.3211	0.2524	0.122*	
C32	0.5319 (6)	0.1666 (4)	0.0742 (2)	0.1061 (15)	
H32A	0.5925	0.2083	0.0382	0.127*	
H32B	0.4158	0.1560	0.0541	0.127*	
H32C	0.5608	0.0873	0.0725	0.127*	

### Atomic displacement parameters (Å^2^)

**Table:**

	*U*^11^	*U*^22^	*U*^33^	*U*^12^	*U*^13^	*U*^23^
N1	0.0301 (9)	0.0400 (10)	0.0312 (10)	0.0074 (7)	−0.0019 (7)	0.0138 (8)
C1	0.0313 (11)	0.0313 (10)	0.0286 (11)	0.0088 (8)	0.0014 (9)	0.0074 (8)
C2	0.0316 (12)	0.093 (2)	0.0397 (14)	0.0075 (12)	0.0007 (10)	0.0285 (14)
C3	0.0246 (10)	0.0375 (11)	0.0286 (11)	0.0064 (8)	0.0043 (8)	0.0128 (9)
C4	0.0326 (11)	0.0319 (10)	0.0302 (11)	0.0097 (8)	0.0035 (8)	0.0117 (8)
C5	0.0314 (10)	0.0290 (10)	0.0267 (10)	0.0076 (8)	0.0049 (8)	0.0078 (8)
C6	0.0261 (10)	0.0321 (10)	0.0251 (10)	0.0058 (8)	−0.0006 (8)	0.0092 (8)
C7	0.0254 (10)	0.0307 (10)	0.0265 (10)	0.0045 (8)	0.0035 (8)	0.0098 (8)
C8	0.0271 (10)	0.0296 (10)	0.0294 (11)	0.0023 (8)	0.0023 (8)	0.0077 (8)
C9	0.0364 (11)	0.0275 (10)	0.0310 (11)	0.0097 (8)	−0.0019 (9)	0.0083 (8)
C10	0.0391 (12)	0.0291 (10)	0.0425 (13)	0.0062 (9)	0.0003 (10)	0.0094 (9)
C11	0.0443 (13)	0.0325 (11)	0.0568 (16)	−0.0004 (10)	0.0009 (11)	0.0073 (11)
C12	0.0585 (16)	0.0284 (11)	0.0561 (16)	−0.0006 (11)	−0.0109 (13)	0.0008 (11)
C13	0.0632 (16)	0.0343 (12)	0.0376 (13)	0.0111 (11)	−0.0010 (11)	0.0009 (10)
C14	0.0451 (13)	0.0336 (11)	0.0321 (12)	0.0119 (9)	−0.0011 (9)	0.0079 (9)
C15	0.0421 (13)	0.0396 (12)	0.0538 (15)	0.0015 (10)	0.0132 (11)	0.0024 (11)
C16	0.107 (3)	0.072 (2)	0.064 (2)	0.0133 (19)	0.0287 (19)	0.0196 (16)
C17	0.063 (2)	0.120 (3)	0.092 (3)	0.037 (2)	0.0111 (19)	−0.012 (2)
C18	0.0510 (14)	0.0400 (12)	0.0347 (12)	0.0113 (10)	0.0077 (10)	0.0051 (10)
C19	0.0736 (19)	0.079 (2)	0.0486 (17)	0.0145 (16)	0.0124 (14)	0.0271 (15)
C20	0.0597 (17)	0.0632 (17)	0.076 (2)	0.0236 (14)	0.0172 (15)	0.0188 (15)
C21	0.0306 (10)	0.0269 (10)	0.0302 (11)	0.0051 (8)	−0.0021 (8)	0.0070 (8)
C22	0.0348 (11)	0.0327 (11)	0.0368 (12)	0.0023 (9)	0.0004 (9)	0.0133 (9)
C23	0.0505 (14)	0.0346 (11)	0.0473 (14)	0.0039 (10)	0.0033 (11)	0.0188 (10)
C24	0.0617 (16)	0.0318 (12)	0.0558 (15)	0.0148 (11)	0.0010 (12)	0.0181 (11)
C25	0.0482 (14)	0.0384 (12)	0.0532 (15)	0.0188 (10)	0.0048 (11)	0.0097 (11)
C26	0.0360 (12)	0.0315 (11)	0.0393 (12)	0.0073 (9)	0.0006 (9)	0.0073 (9)
C27	0.0364 (12)	0.0452 (13)	0.0540 (15)	0.0071 (10)	0.0116 (10)	0.0267 (11)
C28	0.0538 (15)	0.0447 (13)	0.0623 (17)	0.0130 (11)	0.0265 (13)	0.0154 (12)
C29	0.0464 (15)	0.0633 (17)	0.0720 (19)	0.0000 (13)	0.0172 (13)	0.0301 (15)
C30	0.0430 (13)	0.0446 (13)	0.0543 (15)	0.0151 (10)	0.0167 (11)	0.0153 (11)
C31	0.0421 (17)	0.141 (4)	0.144 (4)	0.0071 (19)	0.017 (2)	0.090 (3)
C32	0.157 (4)	0.088 (3)	0.063 (2)	−0.014 (3)	0.046 (2)	0.0058 (19)

### Geometric parameters (Å, °)

**Table:**

N1—C1	1.273 (3)	C18—C19	1.522 (3)
N1—C3	1.419 (2)	C18—C20	1.525 (4)
C1—C1^i^	1.498 (4)	C18—H18	1.0000
C1—C2	1.500 (3)	C19—H19A	0.9800
C2—H2A	0.9800	C19—H19B	0.9800
C2—H2B	0.9800	C19—H19C	0.9800
C2—H2C	0.9800	C20—H20A	0.9800
C3—C4	1.388 (3)	C20—H20B	0.9800
C3—C8	1.397 (3)	C20—H20C	0.9800
C4—C5	1.391 (3)	C21—C22	1.407 (3)
C4—H4	0.9500	C21—C26	1.408 (3)
C5—C6	1.390 (3)	C22—C23	1.392 (3)
C5—C9	1.501 (3)	C22—C27	1.525 (3)
C6—C7	1.396 (3)	C23—C24	1.380 (4)
C6—H6	0.9500	C23—H23	0.9500
C7—C8	1.393 (3)	C24—C25	1.378 (4)
C7—C21	1.498 (3)	C24—H24	0.9500
C8—H8	0.9500	C25—C26	1.395 (3)
C9—C14	1.406 (3)	C25—H25	0.9500
C9—C10	1.405 (3)	C26—C30	1.524 (3)
C10—C11	1.393 (3)	C27—C29	1.529 (3)
C10—C15	1.515 (3)	C27—C28	1.531 (4)
C11—C12	1.377 (4)	C27—H27	1.0000
C11—H11	0.9500	C28—H28A	0.9800
C12—C13	1.379 (4)	C28—H28B	0.9800
C12—H12	0.9500	C28—H28C	0.9800
C13—C14	1.396 (3)	C29—H29A	0.9800
C13—H13	0.9500	C29—H29B	0.9800
C14—C18	1.518 (3)	C29—H29C	0.9800
C15—C17	1.508 (4)	C30—C31	1.504 (4)
C15—C16	1.545 (4)	C30—C32	1.518 (4)
C15—H15	1.0000	C30—H30	1.0000
C16—H16A	0.9800	C31—H31A	0.9800
C16—H16B	0.9800	C31—H31B	0.9800
C16—H16C	0.9800	C31—H31C	0.9800
C17—H17A	0.9800	C32—H32A	0.9800
C17—H17B	0.9800	C32—H32B	0.9800
C17—H17C	0.9800	C32—H32C	0.9800
			
C1—N1—C3	120.55 (18)	C19—C18—H18	107.5
N1—C1—C1^i^	116.3 (2)	C20—C18—H18	107.5
N1—C1—C2	125.69 (18)	C18—C19—H19A	109.5
C1^i^—C1—C2	118.0 (2)	C18—C19—H19B	109.5
C1—C2—H2A	109.5	H19A—C19—H19B	109.5
C1—C2—H2B	109.5	C18—C19—H19C	109.5
H2A—C2—H2B	109.5	H19A—C19—H19C	109.5
C1—C2—H2C	109.5	H19B—C19—H19C	109.5
H2A—C2—H2C	109.5	C18—C20—H20A	109.5
H2B—C2—H2C	109.5	C18—C20—H20B	109.5
C4—C3—C8	120.00 (17)	H20A—C20—H20B	109.5
C4—C3—N1	121.39 (17)	C18—C20—H20C	109.5
C8—C3—N1	118.50 (18)	H20A—C20—H20C	109.5
C3—C4—C5	120.56 (18)	H20B—C20—H20C	109.5
C3—C4—H4	119.7	C22—C21—C26	120.83 (18)
C5—C4—H4	119.7	C22—C21—C7	119.77 (18)
C6—C5—C4	118.94 (18)	C26—C21—C7	119.39 (17)
C6—C5—C9	119.98 (17)	C23—C22—C21	118.4 (2)
C4—C5—C9	121.07 (17)	C23—C22—C27	121.13 (19)
C5—C6—C7	121.37 (17)	C21—C22—C27	120.51 (18)
C5—C6—H6	119.3	C24—C23—C22	121.3 (2)
C7—C6—H6	119.3	C24—C23—H23	119.4
C8—C7—C6	118.96 (17)	C22—C23—H23	119.4
C8—C7—C21	121.08 (17)	C25—C24—C23	120.0 (2)
C6—C7—C21	119.95 (16)	C25—C24—H24	120.0
C7—C8—C3	120.13 (18)	C23—C24—H24	120.0
C7—C8—H8	119.9	C24—C25—C26	121.2 (2)
C3—C8—H8	119.9	C24—C25—H25	119.4
C14—C9—C10	120.88 (19)	C26—C25—H25	119.4
C14—C9—C5	120.06 (19)	C25—C26—C21	118.4 (2)
C10—C9—C5	119.04 (19)	C25—C26—C30	120.0 (2)
C11—C10—C9	118.5 (2)	C21—C26—C30	121.58 (18)
C11—C10—C15	119.7 (2)	C22—C27—C29	113.5 (2)
C9—C10—C15	121.77 (19)	C22—C27—C28	111.90 (19)
C12—C11—C10	120.8 (2)	C29—C27—C28	110.0 (2)
C12—C11—H11	119.6	C22—C27—H27	107.0
C10—C11—H11	119.6	C29—C27—H27	107.0
C11—C12—C13	120.7 (2)	C28—C27—H27	107.0
C11—C12—H12	119.7	C27—C28—H28A	109.5
C13—C12—H12	119.7	C27—C28—H28B	109.5
C12—C13—C14	120.6 (2)	H28A—C28—H28B	109.5
C12—C13—H13	119.7	C27—C28—H28C	109.5
C14—C13—H13	119.7	H28A—C28—H28C	109.5
C13—C14—C9	118.5 (2)	H28B—C28—H28C	109.5
C13—C14—C18	119.5 (2)	C27—C29—H29A	109.5
C9—C14—C18	122.01 (19)	C27—C29—H29B	109.5
C17—C15—C10	112.7 (2)	H29A—C29—H29B	109.5
C17—C15—C16	111.0 (3)	C27—C29—H29C	109.5
C10—C15—C16	109.8 (2)	H29A—C29—H29C	109.5
C17—C15—H15	107.7	H29B—C29—H29C	109.5
C10—C15—H15	107.7	C31—C30—C32	112.0 (3)
C16—C15—H15	107.7	C31—C30—C26	112.7 (2)
C15—C16—H16A	109.5	C32—C30—C26	110.6 (2)
C15—C16—H16B	109.5	C31—C30—H30	107.1
H16A—C16—H16B	109.5	C32—C30—H30	107.1
C15—C16—H16C	109.5	C26—C30—H30	107.1
H16A—C16—H16C	109.5	C30—C31—H31A	109.5
H16B—C16—H16C	109.5	C30—C31—H31B	109.5
C15—C17—H17A	109.5	H31A—C31—H31B	109.5
C15—C17—H17B	109.5	C30—C31—H31C	109.5
H17A—C17—H17B	109.5	H31A—C31—H31C	109.5
C15—C17—H17C	109.5	H31B—C31—H31C	109.5
H17A—C17—H17C	109.5	C30—C32—H32A	109.5
H17B—C17—H17C	109.5	C30—C32—H32B	109.5
C14—C18—C19	112.0 (2)	H32A—C32—H32B	109.5
C14—C18—C20	111.2 (2)	C30—C32—H32C	109.5
C19—C18—C20	111.0 (2)	H32A—C32—H32C	109.5
C14—C18—H18	107.5	H32B—C32—H32C	109.5
			
C3—N1—C1—C1^i^	−178.3 (2)	C11—C10—C15—C17	51.1 (3)
C3—N1—C1—C2	2.1 (3)	C9—C10—C15—C17	−130.1 (3)
C1—N1—C3—C4	71.8 (3)	C11—C10—C15—C16	−73.1 (3)
C1—N1—C3—C8	−112.1 (2)	C9—C10—C15—C16	105.6 (3)
C8—C3—C4—C5	2.0 (3)	C13—C14—C18—C19	−58.5 (3)
N1—C3—C4—C5	178.11 (18)	C9—C14—C18—C19	121.9 (2)
C3—C4—C5—C6	−1.0 (3)	C13—C14—C18—C20	66.3 (3)
C3—C4—C5—C9	177.89 (19)	C9—C14—C18—C20	−113.3 (2)
C4—C5—C6—C7	−0.1 (3)	C8—C7—C21—C22	−83.0 (3)
C9—C5—C6—C7	−179.03 (18)	C6—C7—C21—C22	96.9 (2)
C5—C6—C7—C8	0.2 (3)	C8—C7—C21—C26	98.1 (2)
C5—C6—C7—C21	−179.70 (18)	C6—C7—C21—C26	−82.0 (2)
C6—C7—C8—C3	0.8 (3)	C26—C21—C22—C23	1.5 (3)
C21—C7—C8—C3	−179.31 (18)	C7—C21—C22—C23	−177.34 (19)
C4—C3—C8—C7	−1.9 (3)	C26—C21—C22—C27	−179.36 (19)
N1—C3—C8—C7	−178.08 (18)	C7—C21—C22—C27	1.8 (3)
C6—C5—C9—C14	−89.4 (2)	C21—C22—C23—C24	−0.5 (3)
C4—C5—C9—C14	91.7 (2)	C27—C22—C23—C24	−179.6 (2)
C6—C5—C9—C10	89.2 (2)	C22—C23—C24—C25	−0.7 (4)
C4—C5—C9—C10	−89.7 (2)	C23—C24—C25—C26	0.7 (4)
C14—C9—C10—C11	0.3 (3)	C24—C25—C26—C21	0.4 (3)
C5—C9—C10—C11	−178.33 (18)	C24—C25—C26—C30	−177.4 (2)
C14—C9—C10—C15	−178.52 (19)	C22—C21—C26—C25	−1.5 (3)
C5—C9—C10—C15	2.9 (3)	C7—C21—C26—C25	177.40 (19)
C9—C10—C11—C12	−1.1 (3)	C22—C21—C26—C30	176.3 (2)
C15—C10—C11—C12	177.8 (2)	C7—C21—C26—C30	−4.8 (3)
C10—C11—C12—C13	0.6 (4)	C23—C22—C27—C29	−30.5 (3)
C11—C12—C13—C14	0.6 (4)	C21—C22—C27—C29	150.4 (2)
C12—C13—C14—C9	−1.3 (3)	C23—C22—C27—C28	94.8 (2)
C12—C13—C14—C18	179.0 (2)	C21—C22—C27—C28	−84.3 (2)
C10—C9—C14—C13	0.9 (3)	C25—C26—C30—C31	−57.7 (4)
C5—C9—C14—C13	179.50 (18)	C21—C26—C30—C31	124.6 (3)
C10—C9—C14—C18	−179.45 (18)	C25—C26—C30—C32	68.6 (3)
C5—C9—C14—C18	−0.8 (3)	C21—C26—C30—C32	−109.2 (3)

Symmetry codes: (i) −*x*, −*y*+1, −*z*.
